# Optimization for the production of a polyketone *3S,4S-*DMD from *Panus lecomtei* (Agaricomycetes) by submerged fermentation

**DOI:** 10.1080/21501203.2022.2036842

**Published:** 2022-02-17

**Authors:** Si-Xian Wang, Ping Huang, Hongwei Liu, Yucheng Dai, Xiao-Ling Wang, Gao-Qiang Liu

**Affiliations:** a Hunan Provincial Key Laboratory of Forestry Biotechnology & International Cooperation Base of Science and Technology Innovation on Forest Resource Biotechnology, Central South University of Forestry & Technology, Changsha, China; b State Key Laboratory of Mycology, Institute of Microbiology, Chinese Academy of Sciences, Beijing, China; c Institute of Microbiology, School of Ecology and Nature Conservation, Beijing Forestry University, Beijing, China

**Keywords:** *Panus lecomtei*, (3*S*,4*S*)-3,4-Dihydroxy-6-methoxy-2,2-dimethylchromom, biological activities, submerged fermentation, single factor experiment, Box-Behnken design (BBD)

## Abstract

3,4-Dihydroxy-2,2-dimethyl-chroman derivatives have diverse physiological properties. A polyketone (3*S*,4*S*)-3,4-Dihydroxy-6-methoxy-2,2-dimethylchromom (*3S,4S-*DMD) with antibacterial activity was isolated from the solid culture of rare edible fungus *Panus lecomtei*. However, the yield of *3S,4S-*DMD in solid culture of *P. lecomtei* is very low and the production period are too long. In this work, efficient accumulation of 3*S*,4*S-*DMD in *P. lecomtei* by submerged fermentation is studied. The key fermentation factors of *P. lecomtei* for *3S,4S-*DMD production were optimised by single-factor experiment successively, and then a Box-Behnken design (BBD) experiment was carried out to further enhance *3S,4S-*DMD production. A maximum *3S,4S-*DMD yield of 196.3 mg/L was obtained at 25.78 g/L glucose, 1.67 g/L MgSO_4_ · 7H_2_O, 40°C and 197 r/min, respectively, which increased by 1.3-fold in comparison with that in the non-optimised fermentation conditions. Furthermore, an enhanced yield of *3S,4S-*DMD (261.6 mg/L) was obtained in 5-L agitated fermenter. The *3S,4S*-DMD productivity in flask and fermenter reached to 7.26 and 8.07 mg/g per day, respectively, which considerably increased by over 121-fold in comparison with that in the solid fermentation (0.06 mg/g per day). This study presents a potential method for the production of *3S,4S*-DMD by submerged fermentation.

## Introduction

Natural products and their analogs play a key role in the development of novel drugs because of their enormous structural diversity (David et al. 2015; Newman and Cragg 2020). According to the latest reports of natural products as drug source molecules (Newman and Cragg 2020), over past 40 years, about 65% of the small-molecule drugs on the market are derived from natural products, and mostly are used to against cancer and infection. Due to dual-use characteristics, edible and medicinal fungi have attracted much attention as an important source of drug since ancient times (Wu et al. 2019; Wang et al. 2020). As an important source of nature medicine, secondary metabolism of this kind of fungi has been a hot topic for scientists.

**Table 1.** Factors and levels of Box-Behnken design of fermentation factors of *P. lecomtei.*

Factor	Number	Level		
		−1	0	1
Glucose concentration (g/L)	A	20	25	30
MgSO_4_ · 7H_2_O concentration (g/L)	B	1	1.5	2
Temperature (°C)	C	30	35	40
Shaker speed (r/min)	D	150	200	250

**Figure 1.**
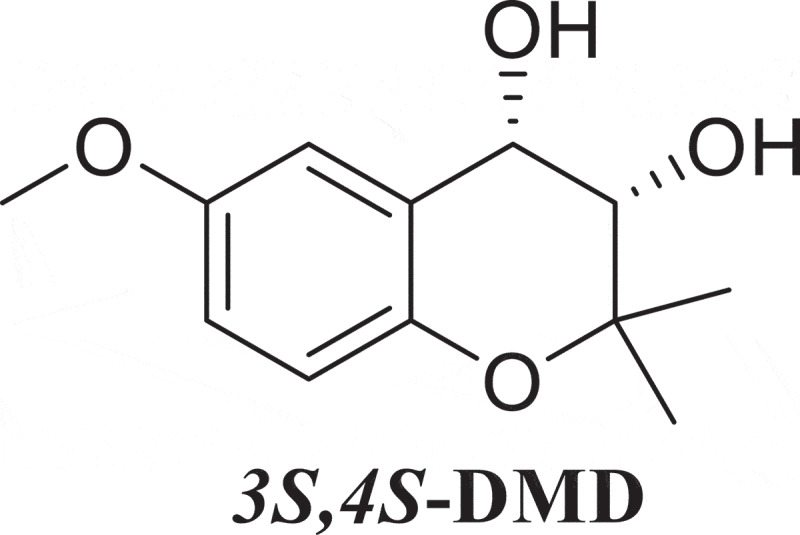
Structure of *3S,4S-*DMD.

*Panus* (Polyporaceae) has been reported to be used as a rare edible and medicine mushroom in parts of Asia and South America (Drechsler-Santos et al. 2012). It is reported to have pharmacological activity on dispersing cold, relaxing tendons, activating collaterals, clearing heat, detoxification, and anti-inflammatory (Wang et al. 2020). The responsible bioactive compounds are polyketone, alkaloid, and sesquiterpene. *Panus lecomtei*, one of the fungi in the genus of *Panus*, is emerging as an edible and medicinal mushroom found in China and the Northern Hemisphere, however, its detailed pharmacological effects and related bioactive compounds are not clear. A recent study reported that the fungus is high in carbohydrate but low in fat, and it showed the presence of phenolics, β-carotene, and lycopene (Sharma et al. 2020). In our recent work, two new meroterpenoid compounds (1 and 2) together with five known meroterpenoid derivatives (3–7) were isolated from solid culture of the fungus. Among which, a polyketone, (3*S*,4*S*)-3,4-Dihydroxy-6-methoxy-2,2-dimethylchromom (*3S,4S-*DMD) (compound 3, Figure 1) was identified (Wang et al. 2020). The compound belongs to 3,4-Dihydroxy-2,2-dimethyl-chroman derivatives, which have diverse physiological properties (Kim et al. 2001).

Our work revealed that *3S,4S-*DMD exhibited antibacterial activity against *Bacillus Calmette – Guérin* (Wang et al. 2020). Furthermore, among the isolated 7 compounds, *3S,4S-*DMD presented the highest yield in the crude extract of solid culture of *P. lecomtei*, suggesting the mushroom strain has the potential to produce *3S,4S-*DMD. However, considering the disadvantage of the long production period (28 days), low yield (735.30 mg in 17.28 g crude extract for 28 days) (Wang et al. 2020), complex process and cannot offer advantages of online monitoring and automation for solid culture (Kumar et al. 2021), in this work, we aimed to produce *3S,4S-*DMD by submerged fermentation of *P. lecomtei*, and further to promote the production of *3S,4S*-DMD by optimisation of culture medium and fermentation conditions with single-factor experiment as well as Box-Behnken design (BBD) experiments. Until now, there are no reports on *3S,4S*-DMD production by submerged fermentation.

## Materials and methods

### Microorganism and strain identification

The strain of *P. lecomtei* was supplied and pre-identified by Dr. Rui-Lin Zhao from State Key Laboratory of Mycology, Institute of Microbiology, Chinese Academy of Sciences.

For further identification of the strain, total DNA was extracted by CTAB method (Huang et al. 2019). Then, 16S rDNA was amplified by PCR to obtain the target fragment. After 1% agarose gel electrophoresis verification, the target fragment was sequenced and identified, and then using BLAST to analyse the ITS sequence in NCBI.

### Preparation of seed broth and fermentation

A few of purified mycelium as the acquisition of inoculum transfer into seed medium (PDB 26 g/L) and incubated at 28°C on a constant temperature oscillation incubator at 180 r/min for 7 days.

Solid cultivation was carried out in 500 mL Fernbach flasks each containing 80 g of rice and 100 mL of distilled H_2_O. Each flask was inoculated with 5 mL seed medium and incubated at 28°C for 28 days (Ahuja et al. 2004).

For submerged fermentation, the strain was cultured in a 500-mL flask containing 180 mL medium at 26°C for 7 days with shaking at 180 r/min.

### Preliminary experiments for submerged fermentation composition

The effects of different carbon sources (glucose, sucrose, maltose), nitrogen sources (beef extract, yeast extract, peptone) and inorganic salts sources (KH_2_PO_4_, K_2_HPO_4_ and MgSO_4_ ·7H_2_O) on the production of *3S,4S-*DMD by *P. lecomtei* in submerged fermentation, and the best fermentation medium composition for *P. lecomtei* was determined.

### Single-factor experiments

The submerged fermentation composition (carbon, nitrogen and inorganic salt), temperature, pH, shaker speed, fermentation time and seed volume were considered as impact factors on the yield of *3S,4S-*DMD from *P. lecomtei*. All these eight factors were firstly estimated by setting 8 factors and 5 levels single-factor experiment (Covino et al. 2010; Ruqayyah et al. 2014).

### Box-Behnken Design (BBD) and Response Surface Methodology (RSM)

According to the results of single-factor experiment, a multi-factor three-level response surface experiment (RSM) was designed (Tables 1, 2). Through the analysis of the multiple fitting equations, the significance results and the response surface graph, the level of influence and the best fermentation scheme among the factors that affect the production of *3S,4S-*DMD by *P. lecomtei* are obtained.

**Table 2.** Box-Behnken experimental design results.

Run	A	B	C	D	Y_Yield_ (g/L)
1	0	−1	1	0	0.2017
2	0	0	0	0	0.2007
3	0	1	−1	0	0.0808
4	−1	1	0	0	0.1257
5	0	0	−1	−1	0.0477
6	0	0	0	0	0.2023
7	−1	0	0	−1	0.0862
8	0	1	0	1	0.1156
9	0	1	0	−1	0.0629
10	−1	0	−1	0	0.1317
11	0	0	0	0	0.2068
12	0	−1	0	−1	0.0856
13	0	1	1	0	0.2160
14	1	−1	0	0	0.1547
15	1	0	−1	0	0.0565
16	0	0	1	1	0.0928
17	1	1	0	0	0.1082
18	1	0	1	0	0.1712
19	−1	−1	0	0	0.1207
20	0	−1	0	1	0.0975
21	0	0	0	0	0.1848
22	−1	0	1	0	0.1386
23	0	0	1	−1	0.1372
24	−1	0	0	1	0.0533
25	1	0	0	1	0.0786
26	0	0	0	0	0.1928
27	0	−1	−1	0	0.1622
28	0	0	−1	1	0.0938
29	1	0	0	−1	0.0258

**Table 3.** Regression coefficients for response surface quadratic model.

Factor	Coefficient Estimate	Standard Error	95% CI Low	95% CI High	VIF
Intercept	0.1975	0.0064	0.1837	0.2113	
A	−0.0051	0.0042	−0.014	0.0038	1
B	−0.0094	0.0042	−0.0184	−0.0005	1
C	0.0321	0.0042	0.0231	0.041	1
D	0.0072	0.0042	−0.0017	0.0161	1
AB	−0.0129	0.0072	−0.0283	0.0026	1
AC	0.027	0.0072	0.0115	0.0424	1
AD	0.0214	0.0072	0.006	0.0369	1
BC	0.0239	0.0072	0.0085	0.0394	1
BD	0.0102	0.0072	−0.0053	0.0257	1
CD	−0.0226	0.0072	−0.0381	−0.0072	1

*R^2^ = 96.59%; *R^2^* (adjust) = 93.18%.

### Bioreactor fermentation

The bioreactor culture was carried out in an agitated 5-L fermenter (New Brunswick Scientific Co., Inc., NJ, USA), under the following conditions: medium volume 3 L, inoculation volume 15% (v/v), temperature 40°C, aeration rate 5.2 vvm, and agitation speed 197 rpm. The concentrations of glucose, yeast extract, MgSO_4_ ·7H_2_O are 25.78, 2.00 and 1.67 g/L, respectively.

### Determination of mycelial biomass

After submerged fermentation of *P. lecomtei*, mycelium was harvested by using a 40-mesh stainless sieve (filter), washed for three times with distilled water, and then centrifugated at 8000 rpm for 15 min, and drying at 60°C until it reached to a constant weight.

### Extraction and measurement of 3S,4S-DMD

The liquid fermented culture (100 mL) or solid fermented substrate (10 g) was dried at 60°C under vacuum, and the solid substances were extracted with 80 mL EtOAc by ultrasonic treatment for 0.5 h (three times), and the organic solvent was evaporated to dryness under vacuum to afford the crude extract, and then it redissolved in methanol for high performance liquid chromatography (HPLC) analysis.

**Figure 2.**
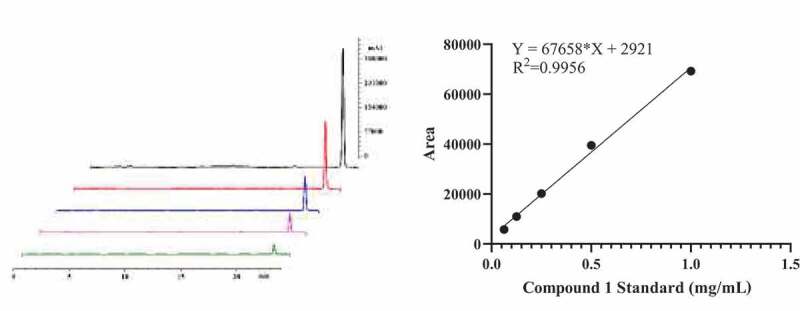
Standard curves of HPLC analysis of *3S,4S-*DMD.

*3S,4S-*DMD was extracted and purified from mycelium of *P. lecomtei* with preparative liquid chromatography in our lab to purities over 98% ($\left[\alpha ]{\;}_{D}^{25}\;-8.4\;(c\;3.14CHCl_{3}\right)$). The quantitative determination, which based on the principle of external standard method (Figure 2), was performed by HPLC (LC-6A, Shimadzu from Japan) with a chromatographic column (C_18_, 25 mm × 4.6 mm, WAT054275, 5 μm, Waters) at a flow rate of 1.0 mL/min^−1^. The mobile phase is water-acetonitrile system, which containing 0.03% trifluoroacetic acid. The elution conditions are 10%–40% acetonitrile for 30 minutes, and then 40%-100% acetonitrile gradient for 10 minutes. The peak area and concentration curve are drawn by GraphPad Prism 8.

### Data statistical analysis

The results of single-factor experiments were analysed by GraphPad Prism 8, and the data of BBD are processed and analysed by Design expert 10 software. All the experiments were conducted in triplicate and the values were expressed as mean. Significant differences were estimated by one-way ANOVA and Duncan’s multiple range tests/student’s t-test.

## Results and discussion

### Strain identification

A total of 652 bases were detected from the obtained ITS sequence of the strains. The BLAST comparison results showed that the two genera with the highest homology are *Panus* and *Lentinus*, with the homology of more than 99%, of which the homology with *Panus lecomtei* hhb-9614 (kp135329.1) is 100%, which proves that the strain is consistent with the previous taxonomic identification results by Dr. Rui-Lin Zhao from Institute of Microbiology, Chinese Academy of Sciences. Therefore, the strain is conformed as *Panus lecomtei.*

### Single-factor experiments of submerged fermentation of P. lecomtei

**Figure 3.**
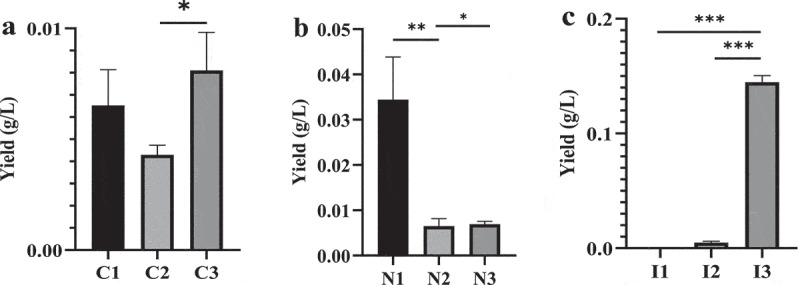
Effects of different carbon sources (a), nitrogen sources (b) and inorganic salts (c) on *3S,4S*-DMD yield in submerged fermentation of *P. lecomtei*. Basic conditions: glucose 25 g/L, yeast extract 2 g/L, MgSO_4_ · 7H_2_O 2 g/L, pH 4.5, temperature 26°C, inoculation amount 10% (v/v), shaker speed 180 r/min, fermentation time 7 days. C1: Glucose; C2: Sucrose; C3: Maltose; N1: Yeast extract; N2: Beef extract; N3: Peptone; I1: KH_2_PO_4_; I2: K_2_HPO_4_; I3: MgSO_4_ · 7H_2_O.

The effects of different carbon sources, nitrogen sources and inorganic salt sources on the production of *3S,4S-*DMD, biomass and EtOAc crude extraction of *P. lecomtei* are shown in Figure 3 and S1. The results showed that when glucose was used as carbon source, the mycelial biomass yield reached the maximum level, and the yield of *3S,4S-*DMD is also higher. Therefore, glucose was selected the suitable carbon source for further study. As shown in Figure 3 and S1, it is obvious that the most suitable nitrogen and inorganic salt sources for both biomass and *3S,4S-*DMD production were yeast extract powder and MgSO_4_ ·7H_2_O, respectively.

* Significant at *p* < 0.05; ** significant at *p* < 0.01; *** significant at *p* < 0.001.

**Figure 4.**
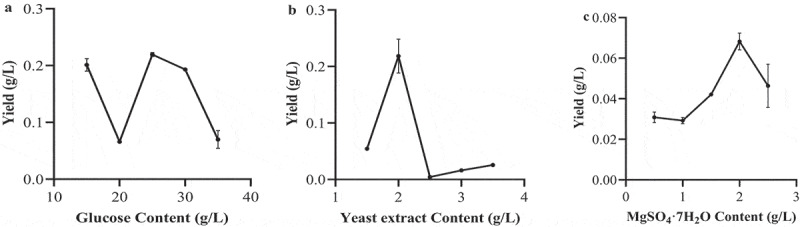
Effects of glucose concentration (a), yeast extract concentration (b) and inorganic salt concentration (c) on *3S,4S*-DMD yield of *P. lecomtei*. Basic conditions: glucose 25 g/L, yeast extract 2 g/L, MgSO_4_ · 7H_2_O 2 g/L, pH 4.5, temperature 26°C, inoculation amount 10% (v/v), shaker speed 180 r/min, fermentation time 7 days.

Figure 4 and S2 show the effects of glucose, yeast extract powder and inorganic salt concentrations on the yield of *3S,4S-*DMD, biomass and EtOAc crude extract. When the glucose concentration was 25 g/L, the yield of crude extract and *3S,4S-*DMD reached the maximum levels, but when it was over 25 g/L, it showed a downward trend (Figure 4(a) and S2a). The reason for this result may be that the concentration of glucose in this medium is too high, which makes the strain’s own osmotic pressure too high, and leads to growth and metabolism inhibition. When the mass concentration of yeast extract reached 2.0 g/L, the yield of biomass, crude extract and *3S,4S-*DMD of the strain reached the maximum levels (Figure 4(b) and S2b). It is interesting that when the yeast extract concentration was 2.5 g/L, the cell growth, especially the *3S,4S*-DMD synthesis was significantly inhibited, and as the concentration of yeast extract increased to 3 or 4 g/L, the yield of *3S,4S*-DMD slightly increased again. This phenomenon occurs may be due to that the components in yeast extract are very diverse and complex. On the one hand, yeast extract can provide nutrients to cells and promote cell to synthetise *3S,4S-*DMD; at the same time, yeast extract may also contain certain components that affect *3S,4S-*DMD synthesis. These components have different effects on *3S,4S-*DMD synthesis at different concentrations. When the concentration of yeast extract is 2.5 g/L, the components that inhibit *3S,4S-*DMD synthesis play a greater role, and when the yeast concentration continues to increase, the concentration of the components that promote *3S,4S-*DMD synthesis increases accordingly, while the components that inhibit *3S,4S-*DMD synthesis do not play a greater role at this concentration than the components that promote *3S,4S-*DMD synthesis, resulting in an increase in *3S,4S-*DMD synthesis. Different concentrations of inorganic salt were added to the liquid fermentation medium, which greatly influenced the crude extract and *3S,4S-*DMD production of this strain (Figure 4(c) and S2c). The optimal inorganic salt concentration for *3S,4S-*DMD production was 2.0 g/L. When the inorganic salt concentration was 1.5 g/L, the biomass was higher than that of other groups, however, the yields of crude extract and *3S,4S-*DMD were not the maximum levels. Therefore, the inorganic salt concentration was selected as 2.0 g/L for further study.

**Figure 5.**
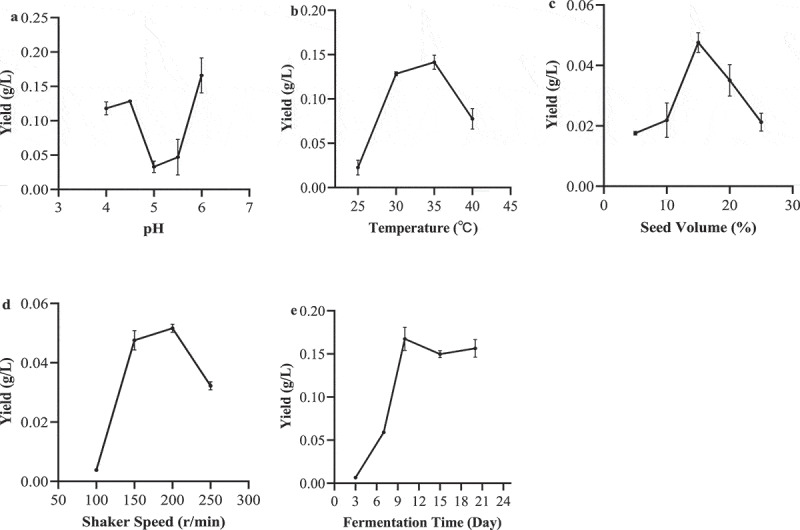
The effects of pH (a), temperature (b), seed volume (c), shaker speed (d) and fermentation time (e) on the *3S,4S*-DMD yield in submerged fermentation of *P. lecomtei*. Medium composition: inorganic salt 2 g/L, yeast extract 2 g/L, glucose 25 g/L.

The effects of initial pH, temperature, seed volume, shaker speed and fermentation time on the biomass, crude extract and *3S,4S-*DMD production are shown in Figure 5 and S3. When the initial pH value was 4.5 or 6.0, the *3S,4S-*DMD yield was at a high level (Figure 5(a)). Considering that the growth environment of the fungus is acidic, therefore, the fermentation pH of this strain was selected to be 4.5. Temperature had a great influence on the yield of *3S,4S-*DMD and biomass, but had less influence on the yield of crude extract. At 35°C, the production of *3S,4S-*DMD and biomass reached the maximum levels (Figure 5(b)). When the seed volume was at 15%, the yields of *3S,4S-*DMD and biomass were the highest (Figure 5(c)) and the yield of crude extract had no significant with seed volume (Figure S3c). When the shaker speed was at 200 r/min, both the yield of *3S,4S-*DMD and biomass were the highest (Figure 5(d) and S3d). After 7 days of fermentation, the biomass production reached the maximum value (Figure S3e), indicating that the viability of this strain was vigorous at this time; the concentration of *3S,4S-*DMD reached the highest level on the 10th day and then decreased (Figure 5(e)). The phenomenon may be because the amount of dissolved oxygen in the fermentation flask continues to decrease with the increase of cell amount, which affects the production of *3S,4S-*DMD. It can also be found from the figure that the fermentation time had no significant effect on the fermentation product. Considering the fermentation efficiency and cell activity, the optimal fermentation time is determined to be 7 days.

Based on the above results, the optimal culture condition was: glucose at 25 g/L, yeast extract at 2 g/L, MgSO_4_ ·7H_2_O at 2 g/L, initial pH at 4.5 g/L, temperature was 35°C, seed volume was 15%, shaker speed at 200 r/min and the fermentation time was 7 days.

### Box-Behnken Design (BBD) and Response Surface Methodology (RSM) results

Four factors and three levels experiments were designed by Box-Behnken design (BBD) method to study the optimal fermentation conditions of *3S,4S-*DMD with higher yield. The experimental results are shown in Table 2 to 4. A second-order polynomial model was generated by applying multiple regression analysis to the experimental data to express the relationship between the independent variables and the response. The final equation obtained in terms of coded factors is given as follows:

(2) $$\begin{array}{rl} & Y_{yield}=0.1975-0.0051A-0.0094B+0.0321C\\ & \;\;\;+0.0072D-0.0129AB+0.027AC+0.0214AD\\ & \;\;\;+0.0239BC+0.0102BD-0.0226CD-0.0525A^{2}\\ & \;\;\;-0.0175B^{2}-0.0177C^{2}-0.0868D^{2} \end{array}$$

**Table 4.** Box-Behnken experimental analysis of variance table.

Source	Sum of Squares	Degree of freedom	Mean Square	*F* Value	*p* value
Model	0.0824	14	0.0059	28.32	< 0.0001
A-Glucose content	0.0003	1	0.0003	1.5	0.2407
B-MgSO_4_ · 7H_2_0 content	0.0011	1	0.0011	5.14	0.0398
C-Temperature	0.0123	1	0.0123	59.36	< 0.0001
D-Shaker speed	0.0006	1	0.0006	2.98	0.1064
AB	0.0007	1	0.0007	3.19	0.0958
AC	0.0029	1	0.0029	13.98	0.0022
AD	0.0018	1	0.0018	8.83	0.0101
BC	0.0023	1	0.0023	11.01	0.0051
BD	0.0004	1	0.0004	2	0.179
CD	0.002	1	0.002	9.85	0.0073
A^2^	0.0179	1	0.0179	86.16	< 0.0001
B^2^	0.002	1	0.002	9.55	0.008
C^2^	0.002	1	0.002	9.75	0.0075
D^2^	0.0489	1	0.0489	235.21	< 0.0001
Residual	0.0029	14	0.0002		
Lack of Fit	0.0026	10	0.0003	3.44	0.1225
Pure Error	0.0003	4	0.0001		
Total deviation	0.0853	28			

where Y_yield_ represents *3S,4S-*DMD yield; A, B, C, D represent glucose concentration, MgSO_4_ ·7H_2_O concentration, temperature, shaker speed, respectively.

As shown in Table 4, the *p*-value of this experimental model is much less than 0.0001, indicating that this model has a high degree of significance (significant at *p* < 0.05, extremely significant at *p* < 0.001), and the lack of fit *p* = 0.1225 > 0.05 indicates that the experimental processes are nearly not affected by other unknown factors. Correlation coefficient *R^2^* = 0.9659 and correction coefficient *R^2^_Adj_* = 0.9318 confirming that the model is reasonable and significant and can better simulate the fermentation process of *3S,4S-*DMD.

Three-dimensional (3D) response surface plots and contour plots visually show the influence of the experimental level of each variable on the yield of *3S,4S-*DMD, and show the optimal level of each variable to obtain the maximum yield of *3S,4S-*DMD. The results are shown in Figure 6 and Table 4. As shown in Figure 6(a,c,e), it was obvious that *3S,4S-*DMD production was sensitive even when the levels of the two factors of each figure were subject to small alterations, and *3S,4S-*DMD yield was high when levels of the two factors of each figure were synchronously in the intermediate range. It can be seen from Figure 6(b) that the yield of *3S,4S-*DMD was high when the glucose concentration was in the intermediate range and temperature was in the higher range of 38–40°C synchronously, and Figure 6(d)and f showed higher temperature leads to higher *3S,4S-*DMD yield. Combined with the above data, the interaction between glucose content and temperature and shaker speed was significant (Figure 6(b,c)), the interaction between temperature and MgSO_4_ ·7H_2_O was significant (Figure 6(d)), and the interaction between temperature and shaker speed was significant (Figure 6(f)), while the interaction between MgSO_4_ ·7H_2_O and glucose content and shaker speed was not significant (Figure 6(a,e)). In addition, the *p*-values of C, A^2^ and D^2^ are all less than 0.0001, indicating that temperature, glucose content and shaker speed have a significant effect on the yield of *3S,4S-*DMD, and the order of the effects is C > D > A > B.

**Figure 6.**
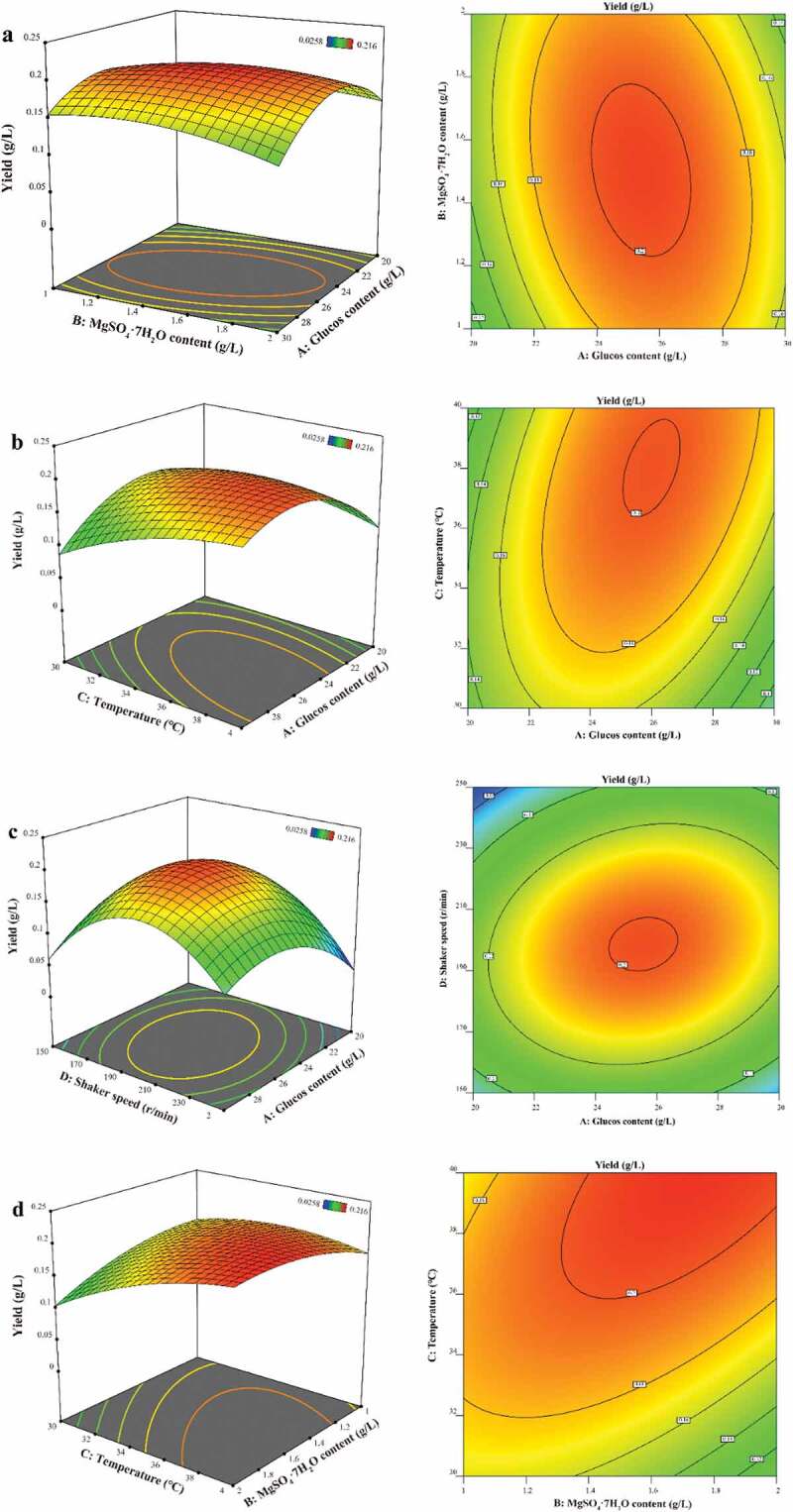
Response surface curve (left) and contour plot (right) for *3S,4S*-DMD (g/L) production illustrating the interaction between any two factors. (**a**) the interaction between glucose content and MgSO_4_ · 7H_2_O concentration; (**b**) the interaction between temperature and glucose content; (**c**) the interaction between shaker speed and glucose concentration; (**d**) the interaction between temperature and MgSO_4_ · 7H_2_O concentration; (**e**) the interaction between shaker speed and MgSO_4_ · 7H_2_O concentration; (**f**) the interaction between shaker speed and temperature.

**Figure 6. Continued:**
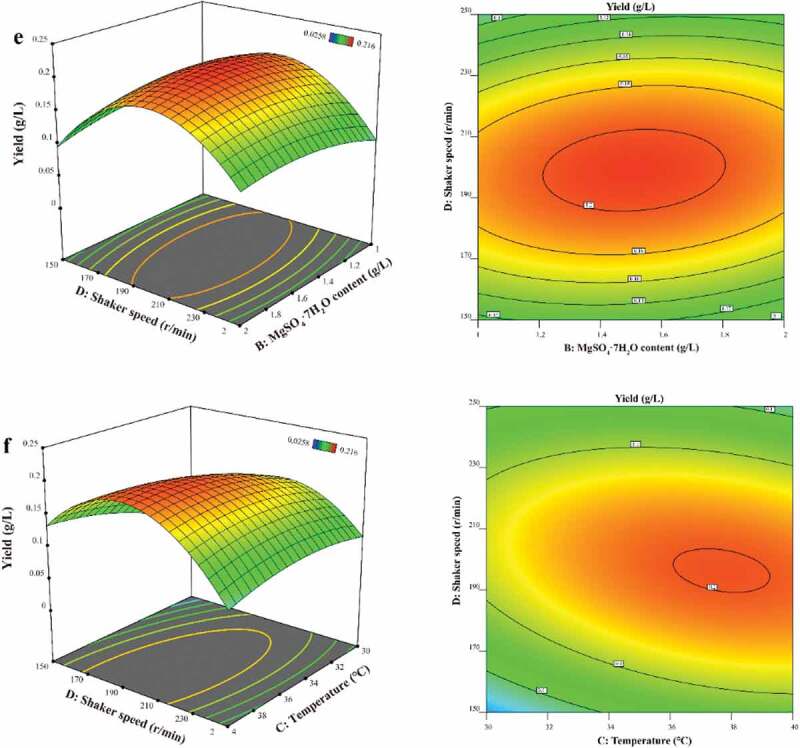

Equation (2) predicts the maximum *3S,4S-*DMD yield of 216.5 mg/L appeared at glucose, MgSO_4_ ·7H_2_O, temperature, rotation speed of 25.78 g/L, 1.67 g/L, 40°C, 197 r/min, respectively.

### Verification of the optimisation model in flask culture and bioreactor culture

The triplicate experiments were carried out to verify the availability and accuracy of the model (Equation (2)) for *3S,4S-*DMD production in flask culture and a 5-L agitated fermenter, respectively. Under the optimum culture condition, 196.3 mg/L of *3S,4S-*DMD was obtained in flask culture, which represented a 126.4% increase in titre compared to the non-optimised culture condition (86.7 mg/L), and was also in agreement with the predicted value (216.4 mg/L). It is noticed that an enhanced yield of *3S,4S-*DMD (261.6 mg/L) was obtained in 5-L agitated fermenter, and a cell density of 3.86 g/L (by dry weight) in flask culture and a cell density of 4.63 g/L in fermenter were obtained, respectively, it could be the reason that the cell growth and product synthesis are more active due to the sufficient oxygen supply in the agitated fermenter. These results also suggest the fermentation condition optimised in the present study was also suitable for *3S,4S*-DMD production in agitated fermenter.

Furthermore, the *3S,4S*-DMD productivity in flask and fermenter reached to 7.26 and 8.07 mg/g per day, respectively (Table 5), which considerably increased by over 121-fold in comparison with that in the solid fermentation (0.06 mg/g per day), suggesting the production of *3S,4S*-DMD by submerged fermentation is a potential method.

**Table 5.** *3S,4S-*DMD production under different fermentation conditions by *P. lecomtei.*

Culture systems	Cell density (g/L)	*3S,4S-*DMD content	*3S,4S-*DMD production (mg/L)	*3S,4S-*DMD productivity (mg/L per day)	*3S,4S-*DMD productivity (mg/g per day)	Culture time
Solid fermentation	-	1.81 (mg/g fermented substrate)	-	-	0.06	28 days
No-optimised Flask liquid fermentation	2.17	39.95 (mg/g mycelium)	86.7	12.39	5.70	7 days
Optimised flask liquid fermentation	3.86	50.85 (mg/g mycelium)	196.3	28.04	7.26	7 days
Optimised bioreactor liquid fermentation	4.63	56.50 (mg/g mycelium)	261.6	37.37	8.07	7 days

For the low yield of many active secondary metabolites, the development of natural drugs is hindered, which has attracted the attention of many researchers. Today there are two main ways to solve this problem, one is through chemical synthesis (Burke and O’Sullivan 1998; Meraz et al. 2016; Schmidt and Riemer 2016), and the other is through fermentation engineering method (Yuan et al. 2008; Gao et al. 2010; Sarchami et al. 2016; Zou et al. 2019) to make the target product high yield. In general, for natural drugs with difficult extraction and low yield, synthetic methods will be chosen to meet production requirements, but this method might cause environmentally unfriendly, excess production capacity and produce many by-products (Ma et al. 2018). In contrast, microbial production is a promising method to produce natural drugs. *P. lecomtei* has been an edible mushroom in China, India and the Northern Hemisphere, particularly it has been used as a medicine in China for several centuries, suggesting it rich in bioactive compounds, however, there is few studies on its detailed pharmacological effects and related bioactive compounds. In our previous work (Wang et al. 2020), we isolated an antibacterial compound *3S,4S-*DMD, but the yield of *3S,4S-*DMD in solid culture of *P. lecomtei* is very low and the production period are too long, in this paper, fermentation optimisation method was successfully used to promote the accumulation of active secondary metabolite (*3S,4S-*DMD) of *P. lecomtei*. The *3S,4S*-DMD productivity in flask and fermenter considerably increased by over 121-fold in comparison with that in the solid fermentation, thus we obtained a feasible method for *3S,4S-*DMD production by submerged fermentation of *P. lecomtei.*

## Conclusion

In this study, the submerged fermentation conditions of *P. lecomtei* for *3S,4S-*DMD production were optimised. The optimal formula of fermentation medium includes 25.78 g/L glucose, 2.00 g/L yeast extract and 1.67 g/L MgSO_4_ ·7H_2_O, and the optimal fermentation conditions are as follows: 7 days fermentation time, 15% inoculation, 197 r/min shaker speed and 40°C. Under these conditions, the yield of *3S,4S-*DMD markedly enhanced to 196.3 mg/L in flask and 261.6 mg/L in fermenter, respectively. Furthermore, the *3S,4S*-DMD productivity in flask and fermenter reached to 7.26 and 8.07 mg/g per day, respectively, which considerably increased by over 121-fold in comparison with that in the solid fermentation (0.06 mg/g per day). This study presents a potential method for the production of *3S,4S*-DMD by submerged fermentation.

## Supplementary Material

Supplemental Material
