# Supplementary material for: Optimization for the production of a polyketone 3S,4S-DMD from Panus lecomtei (Agaricomycetes) by submerged fermentation
Source: Mycology. 2022 Feb 17;13(3):212–22. doi: 10.1080/21501203.2022.2036842 (PMC9354644; doi:10.1080/21501203.2022.2036842)
Supplement: Supplemental Material [file TMYC_A_2036842_SM4560.doc]

Supporting Information (SI)

**Optimization for the production of a polyketone *3S,4S-*DMD from *Panus lecomtei* (Agaricomycetes) by submerged fermentation**

Si-Xian Wang^a^, Ping Huang^a^, Hongwei Liu^b^, Yucheng Dai^c^, Xiao-Ling Wang^*^ and Gao-Qiang Liu^a*^

*^a^Hunan Provincial Key Laboratory of Forestry Biotechnology & International Cooperation Base of Science and Technology Innovation on Forest Resource Biotechnology, Central South University of Forestry & Technology, Changsha, China; ^b^State Key Laboratory of Mycology, Institute of Microbiology, Chinese Academy of Sciences, Beijing, China;* *^c^Institute of Microbiology, Beijing Forestry University, Beijing, China*

Xiao-Ling Wang*, E-mail: wxlcsedu@163.com

*Gao-Qiang Liu, +0731-85623132, E-mail: gaoliuedu@csuft.edu.cn

Supporting Information

[*ITS sequence of* P. lecomtei 3](#__RefHeading___Toc90825167)

[*Figure S1.* ***Effect of different carbon sources, nitrogen sources*** ***and inorganic salt sources*** ***on biomass and crude extract production by*** P. lecomtei in submerged fermentation 4](#__RefHeading___Toc90825168)

[*Figure S2.* ***Effects of glucose concentration, nitrogen concentration*** ***and inorganic salt concentration*** ***on biomass and crude extract production by*** P. lecomtei in submerged fermentation 5](#__RefHeading___Toc90825169)

[Figure S3. The effects of pH (a), temperature (b), seed volume (c), shaker speed (d) and fermentation time (e) on biomass and crude extract production by P. lecomtei in submerged fermentation 6](#__RefHeading___Toc90825170)

# ITS sequence of *P. lecomtei* and BLAST results

**
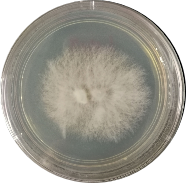
**

CGTTCGGATATGAAGGTTGTAGCTGGCCCTATCCGGGCATGTGCACGCCTTGCTCATTCCAATTCTTACACCTCTGTGCACTTAACATGGGCTGGTCGTAGGCTTTTGTCTTGCTTCACTGTGAGACGGGCTTTGACCTGCCTGTGGTTACTCTACAAACACTTTAAAGTATTAGAATGTAACATCGCGGATAATAAACGCATCTTATACAACTTTCAGCAACGGATCTCTTGGCTCTCGCATCGATGAAGAACGCAGCGAAATGCGATAAGTAATGTGAATTGCAGAATTCAGTGAATCATCGAATCTTTGAACGCACCTTGCGCTCCTTGGTATTCCGAGGAGCATGCCTGTTTGAGTGTCATGGTATTCTCAATTCTCTAAATCTTTGCGGATTTGGATGAATTGGATGTGGAGGTTTATTGCTGGCGACTATCCTTCTGGATCTGTGTCCGGCTCCTCTGAAATAAATTAGCAGGAATGTTGCCGTGCCAACCTCAGTGTGATAATTATCTGCGCTGTTGTTGCTCAGCAAAATATATGTTCTTGCTTCTAATCGTCTTCGGACAATTTCTTGACATCTGACCTCAAATCAGGTAGGACTACCCGCTGAACTTAAGCATATCAAAAAGCGGGAGGAAATTTTTTTTTT


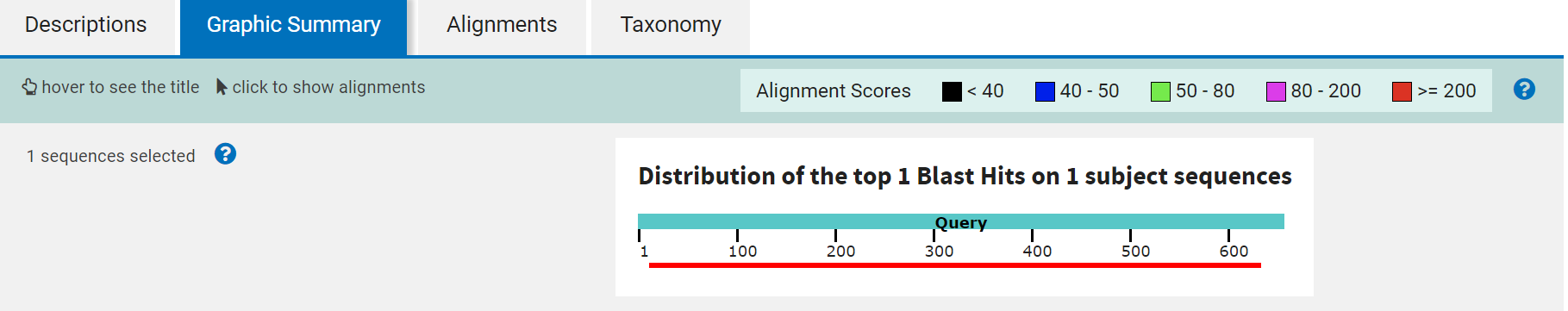

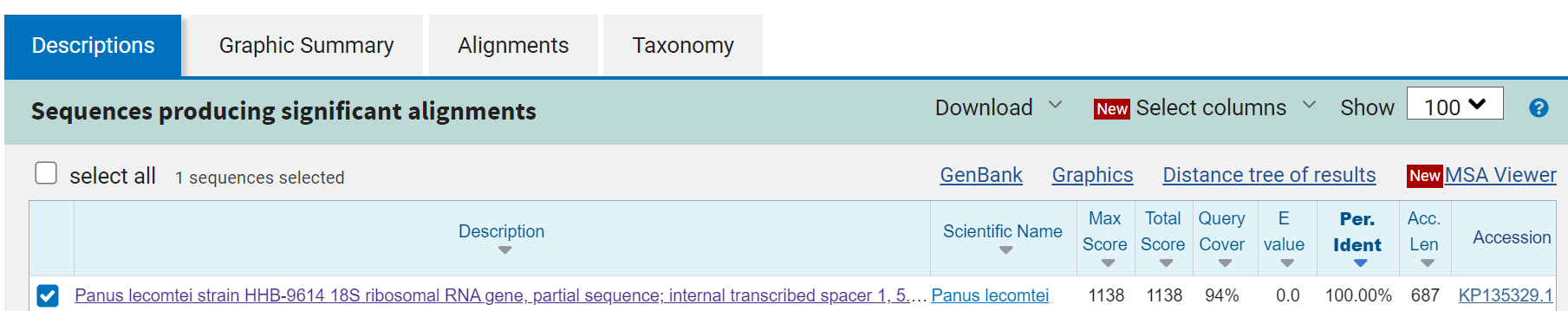


Figure S1. Effect of different carbon sources (**a**, **d**), nitrogen sources (**b**, **e**) and inorganic salt sources (**c**, **f**) on biomass and crude extract production by ***P. lecomtei*** **in submerged fermentation**. Basic conditions: glucose 25 g/L, yeast extract 2 g/L, MgSO_4_·7H_2_O 2 g/L, initial pH 4.5, temperature 26℃, inoculation amount 10% (v/v), shaker speed 180 r/min, fermentation time 7 days. C1: Glucose; C2: Sucrose; C3: Maltose; N1: Yeast extract; N2: Beef extract; N3: Peptone; I1: KH_2_PO_4_; I2: K_2_HPO_4_; I3: MgSO_4_·7H_2_O.

* Significant at *p* < 0.05; ** significant at *p* < 0.01; *** significant at *p* < 0.001.

Figure S2. Effects of glucose concentration (**a**), nitrogen concentration (**b**) and inorganic salt concentration (**c**) on biomass and crude extract production by ***P. lecomtei*** **in submerged fermentation**. Basic conditions: glucose 25 g/L, yeast extract 2 g/L, MgSO_4_·7H_2_O 2 g/L, initial pH 4.5, temperature 26℃, inoculation amount 10% (v/v), shaker speed 180 r/min, fermentation time 7 days.

Figure S3. **The effects of pH (a), temperature (b), seed volume (c), shaker speed (d) and fermentation time (e)** on biomass and crude extract production by ***P. lecomtei*** **in submerged fermentation*.*** Medium composition: inorganic salt 2 g/L, yeast extract 2 g/L, glucose 25 g/L.
